# Traditional Chinese medicine modulates tumor-associated macrophages to suppress gastric cancer progression: mechanisms and therapeutic potential

**DOI:** 10.3389/fonc.2026.1905478

**Published:** 2026-08-20

**Authors:** Xiaodong Zhuang, Qiang Yang, Liangjiu Huang, Risheng Liu, Ting Ma, Liwen Guan, Yana Zheng

**Affiliations:** 1 Department of Clinical Pharmacy, Hainan Cancer Hospital, Haikou, China; 2 Department of Pharmacy, Geriatric Hospital of Hainan Province, Haikou, China

**Keywords:** gastric cancer, TAM reprogramming, therapeutic strategies, traditional Chinese medicine, tumor microenvironment, tumor-associated macrophages

## Abstract

**Background:**

Gastric cancer (GC) remains a leading cause of cancer-related mortality, with progression driven largely by the tumor microenvironment (TME). Tumor-associated macrophages (TAMs), the most abundant immune cells in the GC stroma, drive immune evasion, angiogenesis, metastasis, and chemoresistance through polarization toward an M2-like phenotype.

**Objective:**

This review aims to systematically consolidate the multifaceted molecular mechanisms by which TAMs promote GC aggressiveness and therapeutic resistance, and to categorize the current experimental evidence regarding Traditional Chinese Medicine (TCM) interventions that target TAM reprogramming for anti-GC therapy.

**Methods:**

This narrative review summarizes current evidence retrieved from PubMed, Web of Science, Embase, Scopus, the Cochrane Library, CNKI, Wanfang Data, and the VIP Database from database inception to May 2026, with no language restrictions. The search strategy combined terms related to “gastric cancer” (e.g., gastric cancer, stomach cancer, gastric carcinoma), “tumor-associated macrophages” (e.g., tumor-associated macrophages, TAMs, macrophage polarization, M1 macrophages, M2 macrophages), and “Traditional Chinese Medicine” (e.g., traditional Chinese medicine, Chinese herbal medicine, herbal formula, natural product, active monomer, polysaccharide, terpenoid, alkaloid, catechin), using Boolean operators (AND, OR). Additional relevant studies were identified by manual screening of reference lists of included articles and relevant reviews. Studies were included if they investigated the biological roles of TAMs within the GC TME or the molecular mechanisms by which TCM modulates TAMs, covering both classical herbal formulae and bioactive monomers.

**Results:**

Mechanistically, TAMs promote GC cell proliferation, invasion, and metastasis through the FOXQ1/EMT axis and exosomal ApoE/PI3K/AKT signaling pathways; promote angiogenesis through the VEGF/NF-κB and COX-2/MMP9 signaling pathways; induce resistance to 5-fluorouracil, cisplatin, and oxaliplatin via exosomal miR-21/PTEN, miR-1911-5p, and circ0008253; and mediate immune evasion through the PD-L1- and CLEVER-1-mediated immune checkpoint pathways. Therapeutically, TCM formulae, including Yi-qi-hua-yu-jie-du decoction and Jianpi Yangzheng formula, effectively reprogram TAMs by modulating the PP2A/PI3K/AKT/NF-κB signaling pathway and the exosomal miR-513b-5p/PTEN axis. Active monomers, including Actinidia eriantha polysaccharide, Dendrobium officinale polysaccharide, sophoridine, and epigallocatechin gallate, promote M1-like macrophage polarization and reverse M2-like macrophage-mediated immunosuppression through the PD-1/PD-L1, STAT6/PPAR-γ/JAGGED1/NOTCH1, and TLR4/IRF3 signaling pathways.

**Conclusions:**

TCM-mediated TAM reprogramming represents a promising adjunctive strategy to enhance chemotherapy and immunotherapy efficacy in GC.

## 1 Introduction

Gastric cancer (GC) remains a significant global health concern (1). Recent projections indicate that 15.6 million lifetime GC cases are expected within specific birth cohorts across 185 countries, 76% of which are attributable to *Helicobacter pylori* infection (2). By 2040, the incidence of GC in Asia is projected to surge by over 72%, accompanied by a nearly 76% rise in mortality (3). In China, approximately 358, 700 new cases and 260, 400 deaths were documented in 2022, and the global burden of GC is expected to grow by an additional 62% by 2040, highlighting the enduring clinical challenges associated with this disease (3, 4).

GC progression is increasingly recognized as a consequence of dynamic reciprocal interactions between malignant cells and the tumor microenvironment (TME), rather than a process driven solely by tumor cell-intrinsic mechanisms (5). Within this complex ecosystem, tumor-associated macrophages (TAMs) have been identified as key drivers of GC progression (6, 7). As the most abundant immune population in the GC stroma, TAMs actively coordinate multiple malignant processes, including immunosuppression, angiogenesis, extracellular matrix remodeling, and metastatic spread (8–10). Within the GC TME, TAMs exhibit remarkable functional plasticity and predominantly acquire an immunosuppressive M2-like phenotype, which is closely associated with enhanced epithelial-mesenchymal transition (EMT), lymphangiogenesis, and poor clinical outcomes (11–13). Beyond promoting tumor growth and metastasis, TAMs also contribute to therapeutic resistance by secreting chemokines such as CXCL5 to activate the PI3K/AKT/mTOR signaling pathway, thereby conferring resistance to 5-fluorouracil, and by transferring exosomal miR-21 to suppress PTEN expression, thereby promoting cisplatin resistance (14, 15). Moreover, accumulating evidence indicates that the phenotypic and functional characteristics of TAMs are dynamically shaped through reciprocal interactions with other stromal components. For example, neuronal and cholinergic signaling has recently been identified as an important regulator of macrophage polarization and GC progression (16). Collectively, these findings establish TAMs as promising therapeutic targets for overcoming immune evasion and therapeutic resistance in GC.

Current TAM-directed therapeutic strategies include blocking M2-like TAM recruitment, reprogramming TAMs toward an antitumor M1-like phenotype, and chimeric antigen receptor macrophage (CAR-M) therapy (7, 17). Nevertheless, the complexity of TAM biology, particularly the marked heterogeneity of TAM subsets and the redundancy of signaling networks governing M2 polarization, poses a major challenge to the development of effective targeted therapies (6, 18). In this context, Traditional Chinese Medicine (TCM) presents a promising complementary strategy (19). Meta-analyses have demonstrated that the integration of TCM with chemotherapy significantly improves overall survival and quality of life in patients with GC while maintaining an acceptable safety profile (20). Mechanistic studies have further revealed that TCM formulas can repolarize TAMs from the M2 to the M1-like phenotype by downregulating immune checkpoints such as PI3Kγ, attenuating exosomal miR-513b-5p transfer, and modulating key signaling axes including PI3K/AKT/mTOR and NF-κB/NLRP3 (21–24). Beyond their direct antitumor activity, TCM has long been recognized as adjunctive therapies in oncology because of their ability to enhance the efficacy of chemotherapy and immunotherapy while alleviating treatment-related toxicity and improving patient quality of life. Increasing evidence suggests that these clinical benefits are mediated, at least in part, through remodeling of the TME, particularly via the reprogramming of TAMs (22, 25, 26). Therefore, elucidating the mechanisms by which TCM modulates TAMs may provide a biological basis for integrating the traditional concept of “efficacy enhancement and toxicity reduction” with contemporary precision oncology.

**Figure 1:**
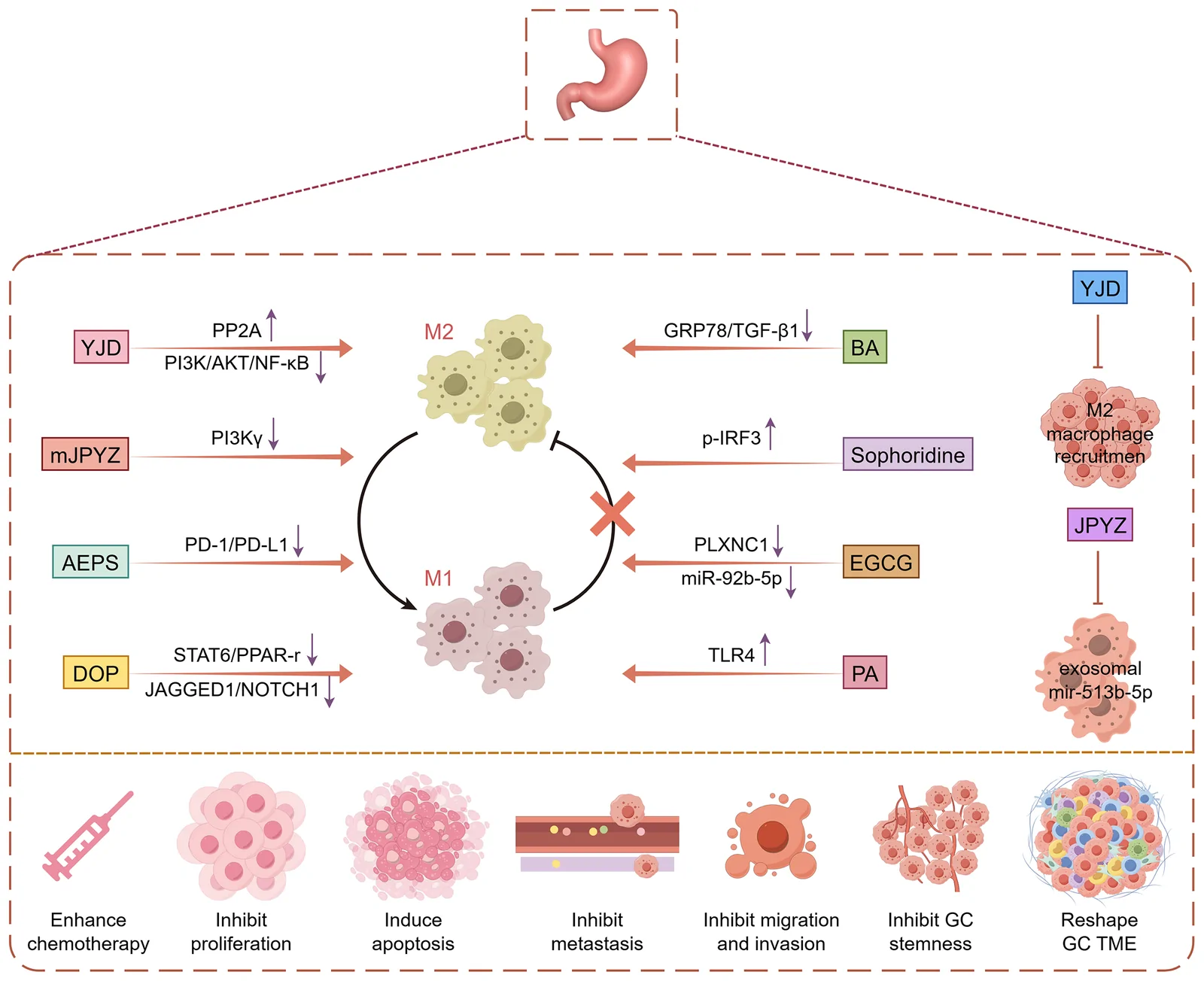
TCM-mediated TAM reprogramming in gastric cancer. TCM formulas (YJD, mJPYZ, JPYZ) and active monomers (AEPS, DOP, BA, sophoridine, EGCG, PA) regulate TAM polarization through multiple signaling pathways, including STAT6/PPAR-γ/JAGGED1/NOTCH1, PI3K/AKT/NF-κB, PD-1/PD-L1, TLR4/IRF3, and GRP78/TGF-β1, as well as exosomal miR-513b-5p and miR-92b-5p. These interventions collectively enhance chemotherapy efficacy, inhibit GC cell proliferation, migration, invasion, metastasis and stemness, induce apoptosis, and reshape the TME.

In this review, we provide a comprehensive overview of the diverse functions and molecular mechanisms of TAMs in GC initiation and progression. We further summarize the current evidence regarding TCM-mediated TAM modulation, including its underlying molecular mechanisms and potential therapeutic targets, with the aim of providing new insights into TAM-targeted therapeutic strategies and a theoretical basis for the integrated management of GC. The effects of these TCM interventions on TAM reprogramming in GC are summarized in **Figure 1**.

## 2 TAMs: biological foundations

TAMs constitute the most abundant immune cell population within the TME, accounting for up to 50% of total stromal cells (27, 28). As key regulators of the tumor immune microenvironment, they serve as an important link between innate and adaptive immunity (29). Therefore, a comprehensive understanding of TAM biology is fundamental to elucidating the mechanisms underlying GC progression and to identifying novel therapeutic targets.

TAMs are predominantly derived from circulating monocytes originating from hematopoietic stem cells (HSCs) in the bone marrow (30). Within the GC TME, chemokines secreted by tumor and stromal cells, particularly C-C motif chemokine ligand 2 (CCL2, also known as MCP-1) and macrophage colony-stimulating factor (M-CSF, also designated CSF-1), recruit circulating monocytes into the TME through interactions with their respective receptors, CCR2 and CSF-1R (31, 32). Following recruitment, these monocytes differentiate into TAMs under the coordinated influence of local cytokines and other microenvironmental cues (33). This recruitment and differentiation cascade underlies the accumulation of TAMs within the GC TME and provides a potential therapeutic target for disrupting TAM-mediated tumor progression.

**Figure 2:**
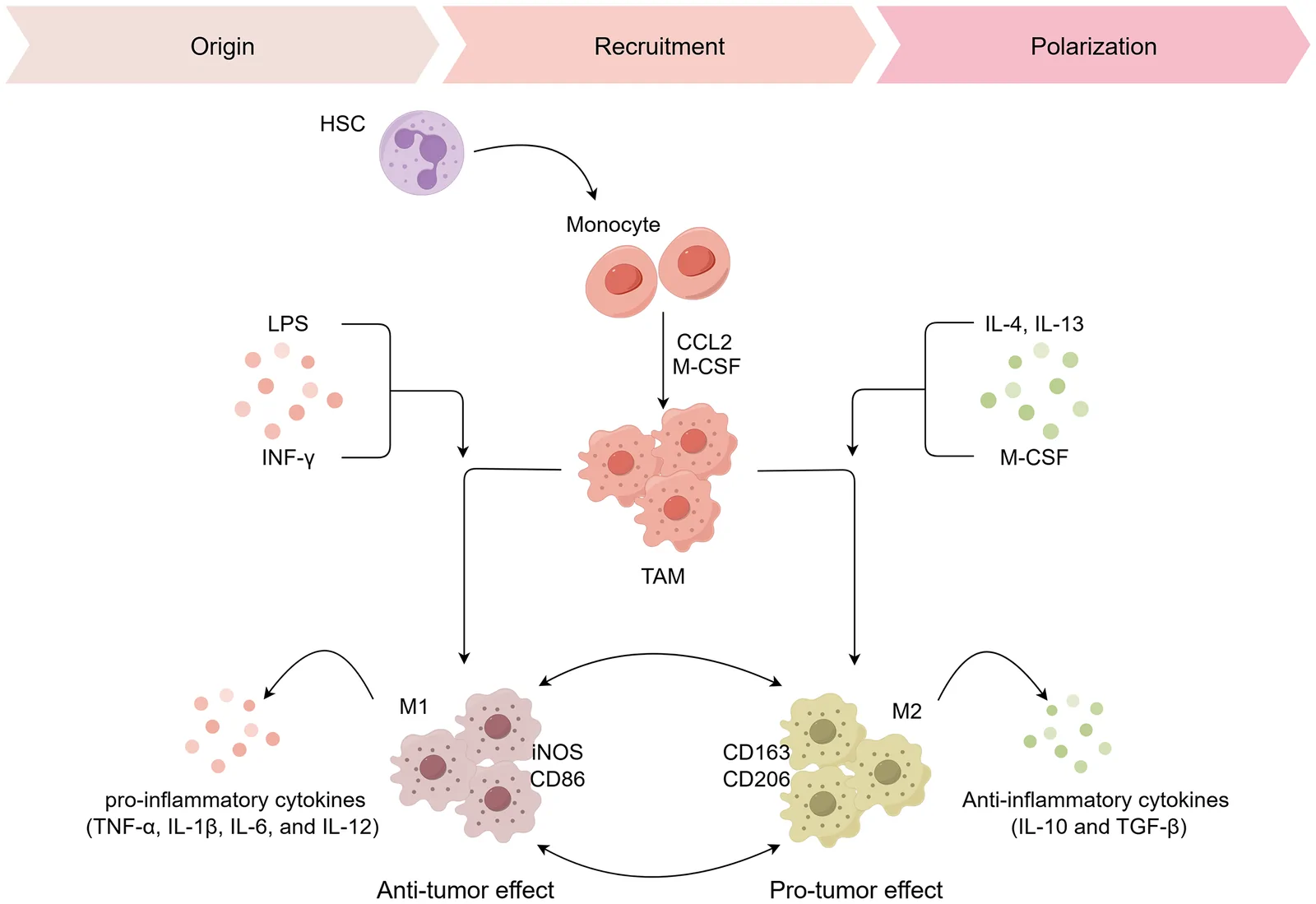
Origin, recruitment, and polarization of TAMs. TAMs originate from hematopoietic stem cells (HSCs) via circulating monocytes. Under the influence of chemokines such as CCL2 and M-CSF, monocytes are recruited into the tumor microenvironment. Upon exposure to LPS/IFN-γ, macrophages polarize into M1-like phenotype (iNOS+, CD86+), characterized by secretion of pro-inflammatory cytokines (TNF-α, IL-1β, IL-6, and IL-12) and exerting antitumor effects. In contrast, stimulation with IL-4, IL-13, or M-CSF drives M2 polarization (CD163+, CD206+), characterized by secretion of anti-inflammatory cytokines (IL-10, TGF-β) and promotion of tumor progression.3. Cancer-Type Specific Applications.

Based on their activation stimuli and functional characteristics, TAMs can be broadly classified into two canonical phenotypes (34). Classically activated M1 macrophages, typically induced by lipopolysaccharide (LPS) or interferon-gamma (IFN-γ), are characterized by high expression of inducible nitric oxide synthase (iNOS) and CD86, production of pro-inflammatory cytokines, including TNF-α, IL-1β, IL-6, and IL-12, and potent antigen-presenting and antitumor activities (35–37). In contrast, alternatively activated M2 macrophages, induced by IL-4, IL-13, or macrophage colony-stimulating factor (M-CSF), are characterized by elevated expression of CD163 and CD206, secretion of anti-inflammatory cytokines, including IL-10 and TGF-β, and functions associated with angiogenesis, tissue remodeling, and tumor progression (38–40). In GC, TAMs predominantly exhibit an M2-like phenotype, largely driven by microenvironmental factors such as hypoxia, lactate accumulation, and chronic inflammatory signaling, which collectively promote an immunosuppressive state (41). Importantly, the M1 and M2-like phenotypes represent two extremes of a dynamic functional spectrum rather than fixed states, with macrophage polarization being continuously reshaped by microenvironmental cues. This remarkable plasticity not only underlies the diverse roles of TAMs in GC progression but also provides a biological basis for TAM-targeted therapeutic interventions (42). The origin, recruitment, and polarization of TAMs within the GC TME are illustrated in **Figure 2**.

## 3 Key mechanisms by which TAMs drive malignant progression in gastric cancer

**Figure 3:**
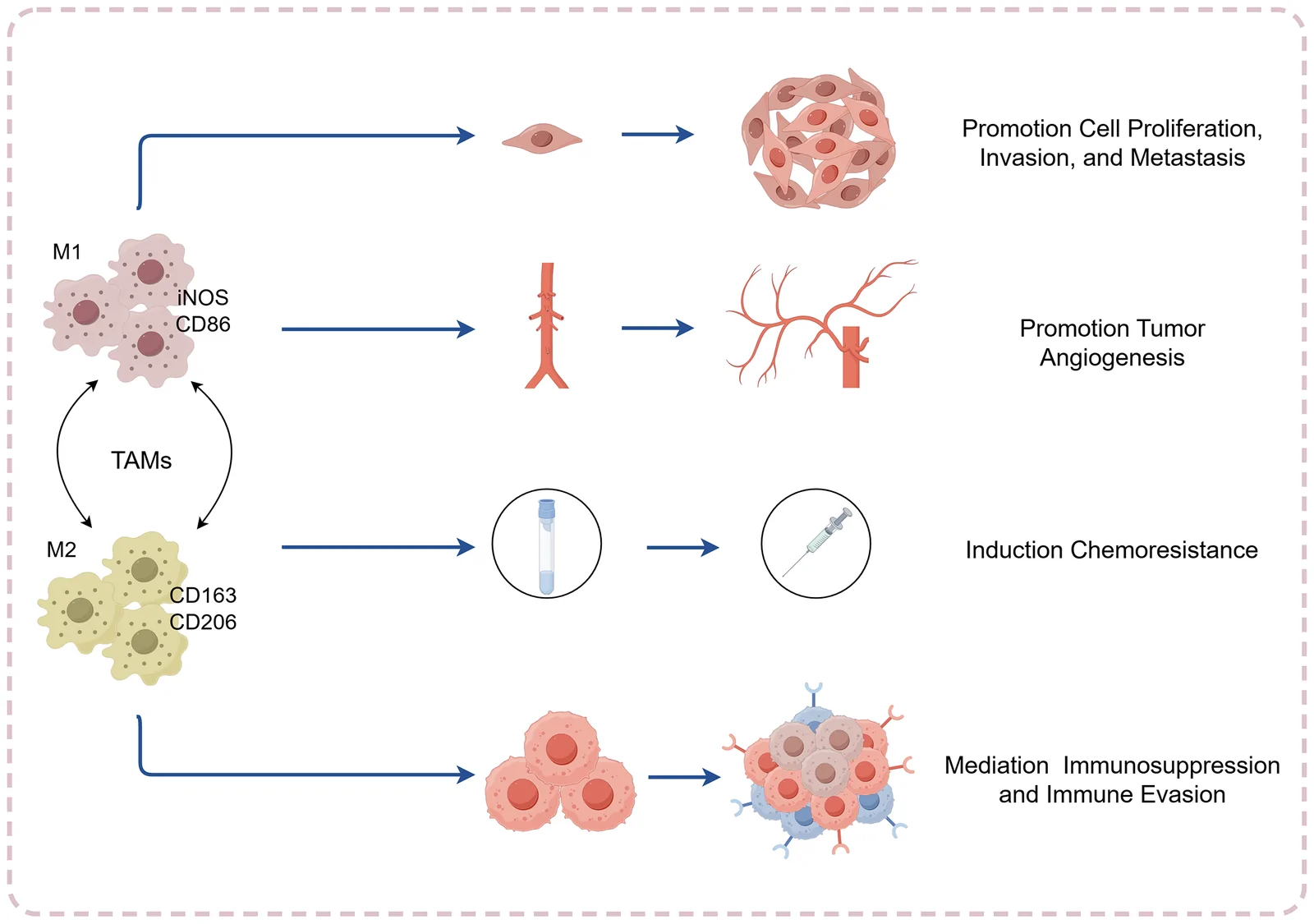
TAM-driven malignant progression in GC. TAMs exhibit functional plasticity, polarizing into M1-like or M2-like phenotypes. M2-polarized TAMs promote GC progression through four major mechanisms: promoting tumor cell proliferation, invasion and metastasis; promoting tumor angiogenesis; inducing chemoresistance; and mediating immunosuppression and immune evasion.

Given that TAMs constitute the predominant immune cell population within the GC TME, they drive tumor progression by regulating multiple biological processes, including tumor cell proliferation, invasion and metastasis, angiogenesis, immune evasion, and chemoresistance, through complex signaling networks and intercellular crosstalk (42). A comprehensive understanding of these diverse functions and their underlying molecular mechanisms is essential for identifying novel therapeutic targets and developing more effective treatment strategies for GC. The major mechanisms by which TAMs drive GC progression are summarized in **Figure 3**.

### 3.1 Promotion of GC cell proliferation, invasion, and metastasis

TAMs promote GC cell proliferation, invasion, and migration through the secretion of a diverse repertoire of cytokines, chemokines, and growth factors. Mechanistically, infiltrating TAMs upregulate forkhead box Q1 (FOXQ1) expression in GC cells (43). FOXQ1, a member of the forkhead box transcription factor family, promotes EMT, as evidenced by reduced E-cadherin expression and increased vimentin expression, thereby markedly enhancing the invasive and migratory capacities of GC cells (43). Moreover, analyses of clinical GC specimens have demonstrated a strong positive correlation between CD68+ TAM infiltration and FOXQ1 expression, highlighting the clinical relevance of the TAM-FOXQ1 axis in GC metastasis (43). Under hypoxic conditions, TAMs activate the CaMKII/ERK1/2/STAT3 signaling pathway, resulting in increased IL-10 secretion, which subsequently promotes GC cell proliferation, migration, and invasion (44). Exosome-mediated intercellular communication further reinforces these protumorigenic effects. Specifically, exosomes derived from M2-like TAMs deliver apolipoprotein E (ApoE) to GC cells, thereby activating the PI3K/AKT signaling pathway and enhancing tumor cell migration (45). Conversely, GC cell-derived exosomal circATP8A1 functions as a sponge for miR-1-3p, activates STAT6 signaling, and promotes M2-like TAM polarization, accompanied by increased secretion of IL-10 and TGF-β, thereby establishing a reciprocal positive-feedback loop between GC cells and TAMs (46).

### 3.2 Promotion of tumor angiogenesis

TAMs actively contribute to neovascularization in GC by secreting a broad spectrum of pro-angiogenic factors. Clinical studies have demonstrated a significant positive correlation between TAM infiltration and microvessel density (MVD) in GC tissues (47). Moreover, TAM abundance is positively associated with preoperative serum levels of VEGF and VEGF-C, highlighting their close relationship with tumor angiogenesis and lymphangiogenesis (48). Mechanistically, co-culture experiments have shown that interactions between TAMs and GC cells markedly increase VEGF and VEGF-C expression, whereas inhibition of the NF-κB signaling pathway significantly attenuates their expression in both cell types, suggesting that NF-κB signaling is a key regulator of TAM-mediated angiogenesis (48). Collectively, these observations indicate that TAMs drive GC angiogenesis and lymphangiogenesis primarily through upregulating VEGF and VEGF-C expression (48). Under hypoxic conditions, TAMs exhibit increased expression of vasohibin-1 and vasohibin-2, both of which are positively correlated with VEGF-A expression, thereby further potentiating angiogenic responses (49). In addition, M2-like TAMs promote angiogenesis and GC invasion by upregulating cyclooxygenase-2 (COX-2), which subsequently enhances matrix metalloproteinase-9 (MMP9) expression (50). Furthermore, single-cell transcriptomics has revealed that SPP1+ TAMs interact with FAP+ cancer-associated fibroblasts and endothelial cells by modulating VEGF signaling, ECM remodeling, and hypoxia pathways, collectively facilitating angiogenesis in GC (51).

### 3.3 Induction of chemoresistance

TAMs are key mediators of therapeutic resistance in GC, primarily through exosome-mediated intercellular communication and the establishment of a protumorigenic microenvironment. In the context of 5-fluorouracil (5-FU) resistance, CXCL5 secreted by M2-TAMs activates the PI3K/AKT/mTOR pathway to promote resistance in GC cells (14). Moreover, GC cells secrete IL-3 to induce M2-like macrophage polarization, which subsequently promotes CCL8 production through GLUT3-mediated glycolytic reprogramming. The released CCL8 further activates the JAK/STAT signaling pathway, forming a feed-forward regulatory circuit that enhances 5-FU resistance (52). In cisplatin-resistant GC, M2-like TAM-derived exosomes transfer miR-21 to GC cells, leading to PTEN downregulation, activation of the PI3K/AKT signaling pathway, and suppression of apoptosis (15). Similarly, exosomal lncRNA CRNDE derived from M2-like TAMs promotes PTEN ubiquitination and degradation, thereby activating PI3K/AKT signaling and further enhancing cisplatin resistance (53). More recently, TAM-derived exosomal miR-1911-5p has been shown to inhibit ferroptosis by targeting the MYB/AKR1B10/ACC axis, while simultaneously promoting M2-like polarization through ARHGEF3 inhibition, thereby establishing a reciprocal positive-feedback loop that further reinforces cisplatin resistance (54). In addition, M2-like TAM-derived exosomes facilitate oxaliplatin resistance by delivering circ0008253 to GC cells, where it suppresses apoptosis and promotes cell survival (55). Beyond conventional chemotherapy, TAMs also contribute to resistance to targeted therapy. Tumor cell-derived microvesicles shuttle glutaminase 1 (GLS1) in a CDC42-dependent manner to promote M2-like TAM polarization, thereby facilitating trastuzumab resistance (56).

### 3.4 Mediation of immunosuppression and immune evasion

TAMs play central roles in GC immune evasion by expressing immune checkpoint molecules and suppressing antitumor T-cell responses through immunoregulatory cytokines. Recent studies have shown that high infiltration of CD74+ TAMs in resectable GC is associated with poor prognosis. Mechanistically, these macrophages establish an immunosuppressive microenvironment through intrinsic PD-L1 expression, whereas PD-1 blockade restores antitumor immunity in CD74+ TAM-enriched tumors (57). Another emerging immune checkpoint molecule, CLEVER-1, is highly expressed on TAMs and correlates with both unfavorable clinical outcomes and reduced responsiveness to anti-PD-1 therapy (58). Mechanistically, CLEVER-1+ TAMs suppress CD8+ T-cell activation and effector function, whereas CLEVER-1 blockade reprograms TAMs toward a pro-inflammatory phenotype, thereby enhancing CD8+ T-cell proliferation and cytotoxic activity (59). Notably, combined blockade of CLEVER-1 and PD-1 further potentiates antitumor immune responses (59). Beyond immune checkpoint regulation, the USP14-IMP2-CXCL2 signaling axis in TAMs promotes resistance to anti-PD-1 therapy by recruiting myeloid-derived suppressor cells (MDSCs), highlighting the multifaceted mechanisms through which TAMs impair immunotherapeutic efficacy (60). In addition, TAMs reinforce the immunosuppressive TME by secreting IL-10 and TGF-β, which suppress CD8+ T-cell function while simultaneously promoting the expansion of regulatory T cells (Tregs) (61).

Collectively, TAMs orchestrate GC progression by promoting tumor cell proliferation, invasion and metastasis, angiogenesis, therapeutic resistance, and immune evasion through multiple interconnected molecular mechanisms. These protumorigenic functions are regulated by diverse signaling pathways, including PI3K/AKT, STAT6/PPAR-γ, NF-κB, TLR4/IRF3, and exosome-mediated regulatory networks, many of which have emerged as promising therapeutic targets. This mechanistic framework provides a strong rationale for TAM-targeted therapeutic strategies and further supports the application of TCM, with its multi-component, multi-target, and multi-pathway characteristics, as a promising approach for TAM reprogramming in GC.

## 4 Traditional Chinese medicine formulas and active monomers targeting TAMs for gastric cancer

Given that TAMs promote GC progression through multiple interconnected biological processes and signaling pathways, overcoming TAM-mediated malignancy remains challenging with conventional single-target therapeutic strategies. By contrast, TCM, characterized by its “multi-component, multi-target, and multi-pathway” mode of action, offers a complementary strategy for TAM reprogramming (62–64). Accumulating evidence has demonstrated that both classical TCM formulas and bioactive monomers modulate TAM function by targeting multiple signaling pathways and regulatory networks implicated in GC progression, thereby providing mechanistic support for their efficacy-enhancing and toxicity-reducing effects in integrated cancer therapy. In this section, we include TCM formulas and active monomers that have been evaluated in GC-related experimental or clinical studies, with direct evidence of TAM modulation and sufficient mechanistic data to support in-depth discussion.

### 4.1 TCM formulas

#### 4.1.1 Yi-qi-hua-yu-jie-du decoction

Yi-qi-hua-yu-jie-du decoction (YJD) is a representative TCM formula that has been clinically applied to reduce postoperative recurrence and metastasis in patients with GC (65). In a prospective cohort of 122 patients with locally advanced GC, the combination of YJD and FLOT neoadjuvant chemotherapy significantly improved the pathological response rate without increasing treatment-related toxicity compared with FLOT alone (65). Mechanistically, YJD remodels the immuno-oncology-microbiome axis by reducing the abundance of *Lactobacillus salivarius*. Given that the abundance of *L. salivarius* is positively correlated with regulatory T cells and M2-like TAMs, these findings suggest that YJD alleviates TAM-mediated immunosuppression through gut microbiota remodeling, thereby enhancing the efficacy of neoadjuvant chemotherapy (65). This mechanism is consistent with accumulating evidence demonstrating that both the gut microbiota and intratumoral microbiota actively regulate macrophage polarization and function (66, 67). Indeed, intratumoral bacteria are increasingly recognized as active modulators of TAM plasticity and tumor progression, highlighting the TCM-microbiota-TAM axis as a promising therapeutic target (68). Beyond microbiota regulation, YJD directly promotes TAM reprogramming by enhancing protein phosphatase 2A (PP2A) activity and suppressing the PI3K/AKT/NF-κB signaling pathway, thereby driving macrophage polarization toward the M1-like phenotype (69). Consistent with these molecular findings, YJD increased the expression of M1-associated markers, inhibited GC cell proliferation, induced apoptosis, and reversed EMT in tumor cell-macrophage co-culture models (69). Furthermore, these antitumor effects were validated in a BALB/c nude mouse xenograft model, in which YJD significantly suppressed tumor growth, reduced M2-like TAM infiltration, and demonstrated a favorable safety profile (69).

#### 4.1.2 Jianpi Yangzheng formula

Jianpi Yangzheng formula (JPYZ) is another representative tonic TCM formula, composed of ten herbs including *Astragalus membranaceus*, *Codonopsis pilosula*, *Atractylodes macrocephala*, *Paeonia lactiflora*, *Citrus reticulata*, and *Angelica sinensis*, and is widely used in clinical GC treatment (70). A modified version of JPYZ (mJPYZ) has been shown to inhibit GC EMT progression through PI3Kγ-dependent TAM reprogramming, ultimately suppressing GC growth and metastasis (21). In addition, JPYZ regulates TAM-derived exosomal communication by reducing miR-513b-5p expression in TAM-derived exosomes, thereby restoring PTEN activity, suppressing aberrant AKT signaling, and ultimately inhibiting GC cell migration and invasion (22). The compositions, sources, and TAM-targeting mechanisms of these TCM formulas are summarized in **Tables 1** and **2**.

**Table 1:** Composition and sources of TCM formulas.

Formula name	Source/origin	Herbal composition
Yi-qi-hua-yu-jie-du Decoction (YJD)	Developed by Professor Liu Shenlin through clinical optimization of the classical formula “Gui Shao Liujun Decoction”, a time-honored prescription historically used to treat spleen-stomach deficiency syndromes with gastrointestinal dysfunction.	• Huangqi (*Astragalus membranaceus*) • Dangshen (*Codonopsis pilosula*) • Baizhu (*Atractylodes macrocephala*) • Danggui (*Angelica sinensis*) • Baishao (*Paeoniae Radix Alba*) • Chenpi (*Citri Reticulatae Pericarpium*) • Banxia (*Pinellia ternat*a) • Sanleng (*Rhizoma Sparganii*) • Ezhu (*Curcuma zedoaria*) • Xianhecao (*Agrimonia pilosa*) • Baihuasheshecao (*Hedyotis diffusa*) • Gancao (*Glycyrrhizae Radix Preparata*)
Jianpi Yangzheng Formula (JPYZ)	Created by Professor Liu Shenlin based on classical texts including “He Shi Xulao Xinchuan” and “Shen Shi Zunsheng Shu”, as the Qi-invigorating and spleen-strengthening component of the Jianpi Yangzheng Xiaozheng (JPYZXZ) decoction.	• Huangqi (*Astragalus membranaceu*s) • Dangshen (*Codonopsis pilosula*) • Sanleng (*Sparganium stoloniferum*) • Ezhu (*Curcuma zedoaria*) • Baizhu (*Atractylodes macrocephala*) • Danggui (*Angelica sinensis*) • Baishao (*Paeonia lactiflora*) • Chenpi (*Citrus reticulata*) • Muxiang (*Vladimiria souliei*) • Gancao (*Glycyrrhiza uralensis*)
Modified Jianpi Yangzheng Formula (mJPYZ)	A further streamlined version of JPYZ based on clinical practice, consisting of four core herbs.	• Huangqi (*Astragalus membranaceus*) • Dangshen (*Codonopsis pilosula*) • Sanleng (*Sparganium stoloniferum*) • Ezhu (*Curcuma zedoaria*)

**Table 2:** TCM Formulas targeting TAMs for GC.

Formula	Model/target	Mechanism/target	Effect	Reference
Yi-qi-hua-yu-jie-du Decoction (YJD)	Locally advanced GC patients	Reshapes gut microbiota (reduces Lactobacillus salivarius abundance); reduces M2-TAM infiltration	Enhances pathological response to neoadjuvant chemotherapy	(65)
Yi-qi-hua-yu-jie-du Decoction (YJD)	Human MKN-45 and AGS cells; GC xenograft mice	Activates PP2A activity; inhibits PI3K/AKT/NF-κB axis; promotes TAM repolarization to M1	Suppresses tumor growth, induces apoptosis, reverses EMT	(69)
Modified Jianpi Yangzheng Formula (mJPYZ)	Human HGC-27 cells; GC xenograft mice	PI3Kγ-dependent TAM reprogramming;	Suppresses GC EMT; inhibits tumor growth and metastasis	(21)
Jianpi Yangzheng Formula (JPYZ)	Human HGC-27 and AGS cells; GC xenograft mice	Reduces TAM-derived exosomal miR-513b-5p; activates PTEN/AKT pathway	Inhibits GC cell migration and invasion	(22)

### 4.2 TCM active monomers

A variety of active constituents derived from TCM have demonstrated anti-GC effects through the modulation of TAM polarization. These studies can be categorized into the following classes. The mechanisms and effects of these active monomers in targeting TAMs are summarized in **Table 3**.

**Table 3:** TCM active monomers targeting TAMs for GC.

Component	Source	Model/target	Mechanism/target	Effect	Reference
Actinidia eriantha polysaccharide (AEPS)	*Radix Actinidia chinensis*	Human AGS cells; GC xenograft mice	Downregulates PD-1/PD-L1 pathway; suppresses M2-type TAM markers; promotes M1 polarization	Inhibits migration/invasion; synergizes with anti-PD-1 antibody	(72)
Dendrobium officinale polysaccharide (DOP)	*Dendrobium officinale*	Human HGC27 and AGS cells; GC xenograft mice	Promotes M2-to-M1 TAM repolarization; modulates EMT markers	Suppresses GC cell migration; induces apoptosis; inhibits tumor growth	(75)
Betulinic acid (BA)	*Birch bark*; *Ziziphus jujuba*	Human AGS cells	Regulates the GRP78-TGF-β1 signaling pathway and macrophage polarization	Inhibits GC stemness	(78)
Sophoridine	*Sophora alopecuroides*	Human MFC cells	Targets TLR4/IRF3 axis; enhances CD8+ T-cell function; suppresses M2-TAMs polarization	Reshape GC TME	(82)
Epigallocatechin gallate (EGCG)	*camellia sinensis*	Human AGS, MKN45 and HGC27 cells	Reverses exosomal miR-92b-5p-induced M2 polarization	Inhibits GC progression	(83)
Palmitic acid (PA)	*Phryma leptostachya*; *Ligustrum lucidum*	Human AGS, and HGC27 cells; GC xenograft	Combined with γ-IFN via TLR4 pathway; Enhances M1-type, reduces M2-type	Inhibits GC cells proliferation/migration and tumor progression	(86)

#### 4.2.1 Polysaccharides

Actinidia eriantha polysaccharide (AEPS), a bioactive polysaccharide isolated from *Radix Actinidia chinensis*, has demonstrated potent antitumor and immunomodulatory activities (71). Both *in vitro* and *in vivo* studies have shown that AEPS significantly suppresses GC cell migration and invasion while reducing tumor growth in xenograft models (72). Mechanistically, AEPS promotes TAM reprogramming toward the M1-like phenotype by inhibiting PD-1/PD-L1 signaling and reducing the expression of M2-associated markers, thereby enhancing antitumor immune responses (72). Furthermore, the combination of AEPS with anti-PD-1 therapy produced superior antitumor efficacy *in vivo*, highlighting its potential as an immunotherapeutic adjuvant for GC treatment (72). Dendrobium officinale polysaccharide (DOP), the major bioactive polysaccharide derived from *Dendrobium officinale*, also exhibits pronounced immunomodulatory and antitumor properties (73, 74). DOP reprograms TAMs from an M2-like to an M1-like phenotype by suppressing the STAT6/PPAR-γ and JAGGED1/NOTCH1 signaling pathways, accompanied by reduced expression of the M2-associated markers ARG1 and TGM2 (75). Consistent with these molecular changes, conditioned medium from DOP-treated macrophages inhibited GC cell migration and promoted apoptosis, whereas DOP administration significantly suppressed xenograft tumor growth and reduced Ki-67 expression *in vivo* (75).

#### 4.2.2 Terpenoids

Betulinic acid (BA), a pentacyclic triterpenoid isolated from the bark of *Betula platyphylla* and seeds of *Ziziphus jujuba*, possesses a broad spectrum of pharmacological activities, including antitumor and anti-inflammatory properties (76, 77). Mechanistically, BA suppresses the expression of stemness-associated proteins in GC cells by inhibiting the GRP78/TGF-β1 signaling axis, while simultaneously preventing GRP78/TGF-β1-mediated macrophage polarization toward the TAM phenotype (78). Consequently, BA disrupts TAM-dependent maintenance of cancer stem cell characteristics, thereby attenuating GC stemness and malignant progression (78).

#### 4.2.3 Alkaloids

Sophoridine, an alkaloid extracted from the seeds of *Sophora alopecuroides*, exhibits multiple pharmacological activities, including antitumor, anti-inflammatory, and antiviral effects (79–81). Mechanistically, sophoridine reprograms TAMs by suppressing M2-like polarization and promoting M1-like polarization through activation of the TLR4/IRF3 signaling pathway. In addition, sophoridine reduces macrophage infiltration by downregulating CCR2 expression and enhances CD8+ T-cell function by alleviating T-cell exhaustion, thereby remodeling the immunosuppressive GC TME (82).

#### 4.2.4 Catechin

Epigallocatechin gallate (EGCG), the major catechin component in *camellia sinensis*, directly interacts with STAT3 to reduce its nuclear localization, promoting transcriptional suppression of PLXNC1, thereby reversing M2-type TAM polarization induced by tumor cell-derived exosomal miR-92b-5p and inhibiting GC progression (83).

#### 4.2.5 Saturated fatty acids

Palmitic acid (PA), a saturated fatty acid extracted from *Phryma leptostachya* and *Ligustrum lucidum*, functions as an endogenous agonist of Toll-like receptor 4 (TLR4) (84, 85). In the presence of interferon-γ (IFN-γ), PA synergistically promotes TAM reprogramming by activating TLR4 signaling, enhancing M1-like polarization while suppressing M2-like polarization. These immunomodulatory effects consequently inhibit GC cell proliferation and migration and suppress tumor growth *in vivo* (86).

## 5 Conclusions and perspectives

TAMs are central drivers of GC malignant progression. Within the GC TME, TAMs predominantly polarize toward an immunosuppressive M2-like phenotype and actively participate in GC initiation, progression, and therapeutic resistance through multiple axes, including promotion of tumor cell proliferation, invasion, and metastasis, induction of angiogenesis, mediation of chemoresistance, and suppression of antitumor immunity. Grounded in the principle of “Reinforce healthy qi to eliminate pathogenic factors”, TCM exhibits unique advantages in targeting TAM reprogramming through its “multi-component, multi-target, multi-pathway” synergistic regulatory paradigm. Importantly, TAM reprogramming provides a mechanistic basis for the well-recognized efficacy-enhancing and toxicity-reducing effects of TCM in cancer therapy by improving antitumor immunity, alleviating immunosuppression, reversing chemoresistance, and potentially enhancing the efficacy of immunotherapy. YJD combined with neoadjuvant chemotherapy has been shown to significantly improve the pathological response rate in locally advanced GC patients, with underlying mechanisms involving gut microbiota remodeling, reduced M2-TAM infiltration, and activation of the PP2A/PI3K/AKT/NF-κB axis to promote M1 polarization. JPYZ and its modified version suppress GC EMT, growth, and metastasis through PI3Kγ-dependent TAM reprogramming, and downregulate miR-513b-5p levels in TAM-derived exosomes to activate the PTEN/AKT pathway, thereby inhibiting GC cell migration and invasion. At the monomer level, AEPS downregulates the PD-1/PD-L1 pathway and promotes TAM polarization toward the M1-like phenotype, and its combination with an anti-PD-1 antibody further potentiates the antitumor effect. Sophoridine promotes M1 polarization via the TLR4/IRF3 axis and enhances CD8+ T-cell function, thereby remodeling the GC TME. These findings provide compelling evidence that TAM-targeted TCM interventions may serve as promising adjunctive strategies to complement conventional treatment modalities, particularly chemotherapy and immunotherapy, by enhancing therapeutic responsiveness and overcoming treatment resistance. Although growing evidence supports the efficacy-enhancing effects of TAM reprogramming, whether it also contributes to the toxicity-reducing effects of TCM during multimodal cancer therapy, including surgery and radiotherapy, remains to be further elucidated. Collectively, these findings not only provide a solid scientific foundation for TCM-targeted TAM strategies in GC therapy but also support the complementary role of TCM in enhancing the efficacy and potentially reducing the treatment-related toxicity of conventional anticancer treatments.

A considerable translational gap remains between preclinical findings and clinical application. Among the interventions reviewed, only YJD in combination with FLOT neoadjuvant chemotherapy has demonstrated clinical evidence with TAM-related mechanistic correlates, whereas all identified bioactive monomers remain at the preclinical stage. Further pharmacokinetic characterization, safety evaluation, and well-designed phase I/II clinical trials are required before their clinical translation. Currently, TCM-mediated TAM modulation should be regarded as a complementary therapeutic strategy rather than a substitute for standard anticancer treatments. Its greatest clinical potential may lie in enhancing therapeutic responsiveness and overcoming TAM-mediated resistance, particularly when integrated with chemotherapy or immunotherapy, which is consistent with current efforts to optimize adjuvant treatment strategies for GC (87). Future prospective studies incorporating TAM subset-specific biomarkers and longitudinal immune monitoring are warranted to facilitate patient stratification and accelerate the clinical translation of TAM-targeted TCM therapies.

In recent years, single-cell RNA sequencing has unraveled the functional heterogeneity of TAMs beyond the classical M1/M2 binary framework, revealing multiple transcriptionally distinct TAM subsets (TAM-APOE, TAM-IDO1, TAM-SKAP1, TAM-POLB) within the GC TME (88). Among these, SPP1+ TAMs interact with exhausted CD8+ T-cells and FAP+ cancer-associated fibroblasts to promote tumor progression and immunotherapy resistance (89). CLEVER-1+ TAMs are associated with poor prognosis and diminished response to anti-PD-1 therapy, fostering an immunosuppressive microenvironment by suppressing CD8+ T-cell function (58). CD74+ TAMs, which are highly infiltrated in resectable GC patients, establish an immunosuppressive microenvironment through autonomous PD-L1 expression and predict poor clinical outcomes (57). Additionally, single-cell RNA sequencing studies have distinguished tissue-resident macrophages from monocyte-derived TAMs, with the latter exhibiting more pronounced protumorigenic functions (90). These findings demonstrate that TAM heterogeneity extends far beyond the conventional M1/M2 classification and highlight the need for precision therapeutic strategies targeting functionally distinct TAM subsets. This emerging paradigm may also facilitate the development of more precise TCM-based interventions aimed at selective TAM reprogramming.

Despite the progress achieved in TCM-targeted TAM research for gastric cancer, many bottlenecks remain. First, this review is confined to herbal formulas and active monomers, which represent pharmacologically active TCM interventions that directly target TAMs through compound-pathway interactions; although TCM also includes non-pharmacological interventions such as acupuncture and moxibustion, direct evidence for their regulation of TAMs in gastric cancer remains limited, and thus they were not included in the present review but warrant further investigation. Second, the evidence specifically demonstrating TCM-mediated TAM regulation in GC remains limited. Most studies are confined to exploratory reports on a small number of formulas or monomers, with systematic mechanistic dissection and target screening severely lacking. Third, single-cell RNA sequencing has revealed at least four functionally distinct TAM subsets within the human GC TME (88). However, the vast majority of studies in the TCM field continue to rely on the simplistic M1/M2 binary classification framework and have not yet developed refined targeting strategies directed at these specific protumorigenic TAM subsets. Fourth, critical translational barriers concerning quality control, pharmacokinetics, and dose-response remain largely unaddressed: batch-to-batch variability and lack of standardized active constituents in herbal preparations compromise reproducibility; the pharmacokinetic profiles and tissue distribution of most active monomers are poorly characterized; and dose-response relationships are rarely established in preclinical studies. Together with the compositional complexity of TCM formulas, these obstacles impede the translation of basic research into clinical application (91). Future research should therefore prioritize the following directions. The functional heterogeneity of TAMs within the GC TME should be dissected using single-cell transcriptomics and spatial transcriptomics to systematically map the dynamic evolution of TAM subsets before and after TCM intervention, thereby identifying TCM-responsive key subsets. Moreover, the “multi-component, multi-target” paradigm requires mechanistic deconvolution. Network pharmacology and AI-driven strategies can identify active drivers of TAM reprogramming, resolve synergistic contributions, and exclude non-specific effects, and data-mining of prescription rules further complements this approach (92–94). Concurrently, multicenter, randomized, double-blind, placebo-controlled trials employing parameters such as the M2/M1 ratio, proportions of specific TAM subsets, and exosomal biomarkers as biological endpoints are urgently needed to validate the clinical efficacy of TCM in TAM reprogramming. Integrating multi-parametric clinical and biomarker data into decision-support frameworks will be essential to guide patient stratification and regimen selection—for example, using CD74+, CLEVER-1+, or M2/M1 ratio as biomarkers to identify patients most likely to benefit from specific TCM-TAM interventions. Given that TAM-driven PD-L1 expression and immunosuppression are central to immune checkpoint inhibitors resistance, TCM-induced M1 repolarization may synergistically enhance anti-PD-1/PD-L1 efficacy. However, whether TCM-mediated TAM reprogramming can consistently sensitize tumors to immune checkpoint blockade remains to be systematically investigated.
